# Competitive influence maximization and enhancement of synchronization in populations of non-identical Kuramoto oscillators

**DOI:** 10.1038/s41598-017-18961-z

**Published:** 2018-01-15

**Authors:** Markus Brede, Massimo Stella, Alexander C. Kalloniatis

**Affiliations:** 1 University of Southampton, Electronics and Computer Science, Southampton, SO171BJ UK; 2 Fondazione Bruno Kessler, Trento, Italy; 3 Joint and Operations Analysis Division, Defence Science and Technology Group, Canberra, 2600 Australia

## Abstract

Many networked systems have evolved to optimize performance of function. Much literature has considered optimization of networks by central planning, but investigations of network formation amongst agents connecting to achieve non-aligned goals are comparatively rare. Here we consider the dynamics of synchronization in populations of coupled non-identical oscillators and analyze adaptations in which individual nodes attempt to rewire network topology to optimize node-specific aims. We demonstrate that, even though individual nodes’ goals differ very widely, rewiring rules in which each node attempts to connect to the rest of the network in such a way as to maximize its influence on the system can enhance synchronization of the collective. The observed speed-up of consensus finding in this competitive dynamics might explain enhanced synchronization in real world systems and shed light on mechanisms for improved consensus finding in society.

## Introduction

Understanding the interplay between topology and dynamics on complex networks is one of the fundamental problems in complex systems science. One area, in which much progress has been made in recent years, is studying the dynamics of synchronization of chaotic or limit cycle oscillators on complex networks, which has many applications in the biology of interacting neurons or heart cells as well as social or technical systems in which people, lasers or power systems have been observed to produce collective rhythms^1–4^. Of some importance for the interpretation of the results below is also that models of coupled oscillators have also been proposed to explore consensus finding in society^5,6^.

Insights gained have revolved around the impact of network topology on synchronization, i.e. finding that adding long range connections in spatial systems can enhance synchronization^7,8^, or observations that the synchronization transition is influenced by network heterogeneity on scale-free networks^9,10^. Other research has investigated the impact of modularity on time scales to synchronization^11^, weighting schemes that can enhance synchronization properties of networks^12^, or works that reveal different paths to synchronization on different types of networks^13^. More recently there has been interest in adaptive coupling schemes whose co-evolution with oscillator dynamics can boost synchronization. In this area the focus has often been on adaptation rules that allow for rewiring which only use local information, e.g. demonstrating that coupling which grows in proportion to phase or state differences^14,15^ or rewiring towards more out-of-phase oscillators^16^ can enhance synchronization, or that forming connections between oscillators with similar phases can lead to a concurrent enhancement of percolation and synchronization^17^.

A prominent question in the above research is to find “optimal” networks for synchronization^12,14,18–24^. This issue gains an additional dimension of complexity when oscillators are not identical, such that correlations between oscillator type and topological position can influence the dynamics of synchronization. In this context it has been found that anti-correlated placements of oscillators on networks can enhance synchronization^19,21,22,24^ or lead to first order transitions on heterogeneous networks^25^. In the last few years these findings have been understood via a collective coordinate approach^24,26^. Very recently also the game theory of synchronization was considered, exploring the influence of agents’ choices between making an effort to get synchronized or free-riding and waiting for others to synchronize to them^27^. The latter study is motivation to our work, but we focus on the network game of agents attempting to maximize their influence on the collective instead.

So far, most optimization schemes for complex networks have considered how a central planner can design a system to optimally achieve a certain target^18,20,23,28^. In some complex systems a scenario of such central control does not apply. Instead, system structure may arise as a result of individuals striving to better systemic outcomes from their individual perspectives. Fewer studies have analyzed this game-theoretic situation in which nodes compete to structure a network to achieve individual targets which need not necessarily be aligned with the best trade-off at the system level. This is, for instance, the case for synchronization, where node specific aims of achieving best local synchronization may be in conflict with global synchronization^29^. Here, we consider a variant of this problem, in which individual nodes of a directed network attempt to rewire connections in order to maximize their influence on the collective such that they drag the frequency of collective synchronization towards their respective preferences. One might anticipate that such a system can never achieve global consensus or dynamic equilibrium. However, as elaborated below, we find that under certain conditions such individual rewiring aimed at alignment of the system with individually very diverse node-specific goals actually tends to accelerate global consensus finding, e.g. synchronization.

## Model

Consider a system of *N* coupled oscillators with phases *ϕ*_*i*_ and native frequencies *ω*_*i*_ whose dynamics are specified by the well-known Kuramoto dynamics^30^

1 $${\dot{{\varphi }}}_{i}={\omega }_{i}+\kappa \sum _{j}{a}_{ij}\,\sin \,({{\varphi }}_{j}-{{\varphi }}_{i}),$$

where the adjacency matrix of the coupling network has the properties *a*_*ij*_ = 1 if there is a link from *j* to *i* and *a*_*ij*_ = 0 otherwise, while *κ* denotes the coupling strength. The native frequencies *ω*_*i*_ are drawn from some distribution, e.g. the uniform distribution between [−1, 1]. Below, we will assume that a node’s native frequency represents the nodes frequency preference. Depending on the structure of the coupling network and the coupling strength, the Kuramoto model on complex networks can exhibit a phase transition at some critical coupling *κ*_*c*_ such that a macroscopic fraction of the oscillators are phase and frequency locked for *κ* > *κ*_*c*_ while this is not the case below the critical coupling. Conventionally one defines the order parameter *r*

2 $$r=\mathrm{(1/}N)|\sum _{j}\,\exp \,(i{{\varphi }}_{j})|,$$

such that *r* ≈ 0 indicates lack of synchronization while values of *r* close to one indicate a highly synchronized state.

Summing over Eq. (1) shows that for symmetric coupling/undirected coupling networks *a*_*ij*_ = *a*_*ji*_ synchronization will always occur to the mean of the ensemble of native frequencies. However, it is important to note that this is generally not the case for directed coupling in which the collective (dynamical) frequency can be influenced by the structure of the coupling network. Exactly this is the basis of the model below in which we consider a set of self-interested Kuramoto agents which attempt to rewire connections aiming to align collective behavior (i.e. the collective frequency) with their individual goals (i.e. their respective native frequencies). In principle, this problem constitutes a network formation game^31^. However, the involved non-linearities make an analytical calculation of equilibria for systems of interesting size unfeasible. Instead, in the spirit of Brede^28^ we consider a dynamics in which agents subsequently attempt to approach their aims by local rewiring.

**Figure 1:**
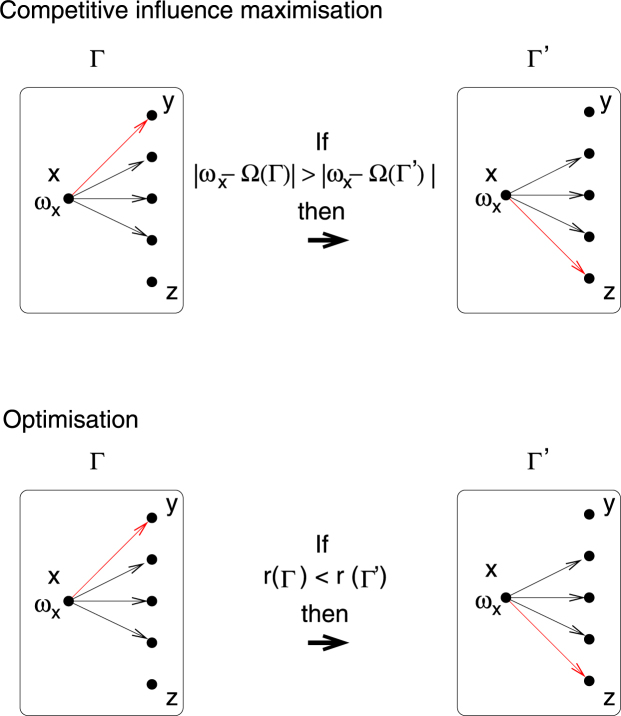
Illustration of rewiring to maximize influence on the collective or conventional optimization. In the first, rewiring to new nodes is accepted if it drags the collective frequency Ω close to the nodes’ own native frequency. In the second, rewiring is accepted if it enhances the degree of global synchronization measured by the order parameter *r*.

More precisely, consider a set of *N* nodes each of which disposes of *k*_out_ links with which to influence the collective dynamics. After initially assigning connections at random (thus generating a random graph which is regular in out-degree), nodes then iterate the following process: (i) a random focus node *x* is chosen and one of the out-connections of the focus node, say *x* → *y*, is selected at random and a rewiring of this connection to a random target node *z* is considered; (ii) node *x* will change from influencing *y* to influencing *z* if the respective rewiring will result in a collective frequency of the ensemble that is closer to *x*’s native frequency than the collective frequency of the ensemble with the link *x* → *y* in place. If this is not the case, the rewiring is not performed and the network remains unaltered. Steps (i) and (ii) are iterated until some quasi-stationary equilibrium is reached. For comparison, we also consider a conventional optimization scheme in which links *x* → *y* are rewired to *x* → *z* if the respective rewiring results in a more synchronized system state. See Fig. 1 for a schematic illustration of the two rewiring schemes. Note that in both schemes the distribution of out-degrees remains regular through-out the procedure – each node has the same potential to influence the system – while the in-degrees will change.

In the following we consider a numerical implementation of the above scheme. To estimate collective frequencies of the oscillator ensemble, numerical integration of Eq. (1) is performed using a Runge-Kutta 4th order method with step size 0.1 over a time interval [0, *T*] where we usually set *T* = 1000 which is long enough to reach equilibration after *T*/2. After discarding an initial transient up to *T*/2 collective frequencies are then estimated by ${\rm{\Omega }}=\mathrm{1/}N{\langle {\sum }_{i}{\dot{{\varphi }}}_{i}\rangle }_{[T\mathrm{/2,}T]}$, where 〈 ⋅ 〉_[*T*/2,*T*]_ stands for a time average over the interval [*T*/2, *T*]. We have experimented with different system sizes of systems up to *N* = 500 oscillators and different integration steps, but for reasons of limited computation time we only report qualitatively robust results for networks of *N* = 100 oscillators below. Since rewiring in our model is based on node frequencies, we also measure frequency synchronization via

3 $${\rm{\Delta }}f={{\rm{\sigma }}}_{{\langle \dot{{\varphi }}\rangle }_{[T\mathrm{/2,}T]}}/{{\rm{\sigma }}}_{\omega },$$

which measures the ratio of the standard deviations *σ* of the distribution of the average instananeous frequencies ${\dot{{\varphi }}}_{i}$ over the period [*T*/2, *T*] relative to the standard deviation of native frequencies. Thus Δ*f* = 0 indicates perfect frequency synchronization and Δ*f* ≈ 1 corresponds to a state without frequency synchronization.

## Results

### Rewiring dynamics

**Figure 2:**
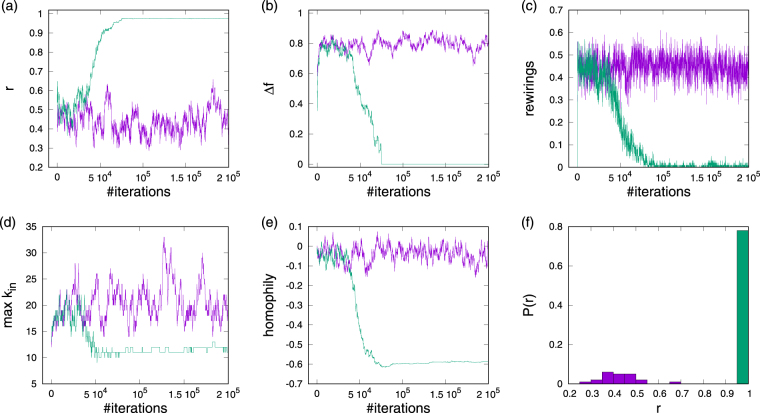
Evolution and statistics of synchronization properties during rewiring for sample configurations that evolve for *κ* = 0.23. Green lines give an example of a typical synchronized and purple lines an example of a typical non-synchronized configuration. The panels show: (**a**) order parameter *r* for phase synchronization, (**b**) order parameter Δ*f* for frequency synchronization, (**c**) fraction of accepted rewiring per 100 attempts, (**d**) maximum in-degree, (**e**) homophily of native frequencies, and (**f**) distribution of order parameters for equilibrated configurations. Systems are of size *N* = 100 and *k*_out_ = 6 and *κ* = 0.23 was used. The distribution (**f**) is estimated from 100 experiments.

By plotting some key system properties as a function of the number of rewiring iterations, Fig. 2 illustrates typical dynamics of the competitive influence-maximization for some specific system configurations for a network of *N* = 100 nodes, out-degree *k* = 6 and coupling strength *κ* = 0.23. The choice of a fixed out-degree reflects the idea that there is a limit to how many separate nodes can be influenced by any one agent in the system. For that level of coupling in random configurations typically partial synchronization is found corresponding to an order parameter of *r* ≈ 0.58. Then, starting the competitive rewiring process from this low level of synchronization essentially two possible outcomes are observed. First, for some starting configurations of networks and native frequencies global synchronization initially declines and then fluctuates around some low level concomitant with ongoing fluctuations in the average instantaneous frequency Ω. At the same time, the rewiring dynamics remains active with about 45% of suggested rewirings being accepted (Fig. 2c), and no global consensus is found, even after very large numbers of iterations. In these cases influence maximization results in the formation of a core-periphery network which we will discuss in more detail below. Second, for some other initial conditions, concurrent enhancement of synchronization and influence maximization is found. In these cases the system always ends up in an almost fully synchronized state in which all nodes have been recruited into a frequency locked cluster. The saturation towards this state goes hand in hand with a drop off in rewiring activity so that eventually a static network configuration is approached, i.e. an equilibrium is reached that is stable in the sense of not being unilaterally changeable by the requirements of individual nodes. The discrete nature of possible outcomes is illustrated in panel (f) in which we plot the distribution of final values of order parameters estimated from a sample of 100 independent simulation runs. One notes that this distribution is clearly bimodal with peaks around *r* ≈ 0.35 and *r* ≈ 0.98 marking non-synchronized and synchronized networks. As we will explore in more detail later, the high synchrony peak quickly grows to domination when larger coupling strengths *κ* > 0.23 are chosen such that, e.g., for *κ* = 0.27 all 100 evolved configurations grow to full synchronization. Interestingly, such configurations do not only show enhanced synchronization for the coupling strength at which they were evolved, they also show markedly improved synchronization levels for a large range of coupling strengths and an onset of macroscopic synchronization for much lower coupling than random networks, c.f. Fig. S1 in the Supplementary material.

We also see that for synchronizing and non-synchronizing trajectories the network topology is affected in different ways. To illustrate this and follow the dynamics of network change, we monitor two characteristics of network organization. First, to get an impression of changes in the degree distributions, panel Fig. 2d shows the maximum in-degree. Large fluctuations around a mean value distinctly above the average for random configurations are found for non synchronized situations, whereas after an initial transient a reduction in degree heterogeneity is observed when convergence towards synchronization occurs. In panel Fig. 2e we plot the strengths of correlations between linked native frequencies measured by Newman’s homophily coefficient^32^. Saturation towards strong anti-correlations occurs when approaching full synchronization, and fluctuations about a slightly negatively anti-correlated native frequency arrangement are found in the opposite case.

### Network organization

**Figure 3:**
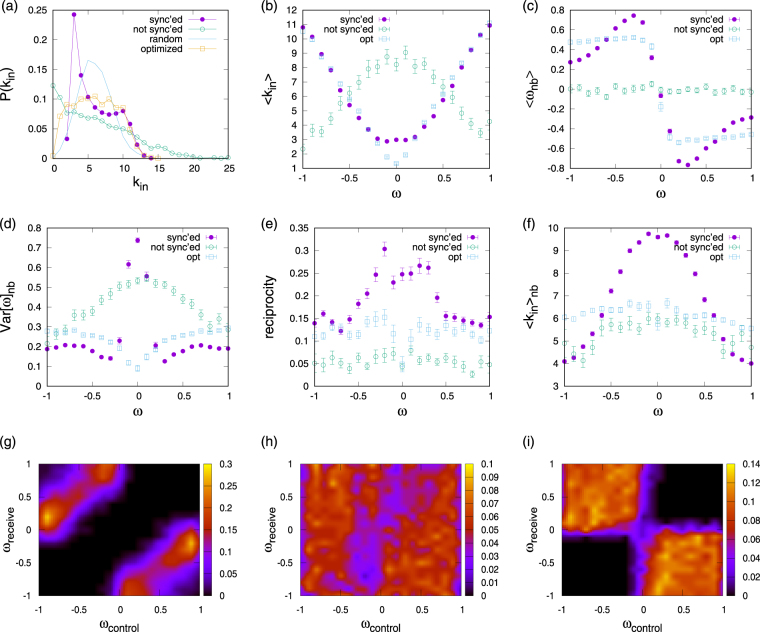
Network analysis for competitively evolved and optimized networks: (**a**) in-degree distribution, (**b**) dependence of average native frequency on degree, (**c**) dependence of average native frequency of in-neighbors on native frequency, (**d**) dependence of variance between native frequencies of in-neighbors on native frequency, (**e**) dependence of reciprocity on native frequency, (**f**) dependence of in-degree of in-neighbors on native frequency (hinting to a strongly disassortative arrangement: *a* = −0.73 ± 0.06, *a* = −0.19 ± 0.05 (opt), *a* = −0.16 ± 0.04 (not sync’ed), *a* = −0.04 ± 0.03 (random). Illustrations of linked *ω* − *ω* correlation matrices for competitively evolved synchronized (**g**), non-synchronized (**h**) and conventionally optimized (**i**) networks. Configurations evolved for *κ* = 0.23 and *N* = 100.

In fact, synchronized and non-synchronized networks evolve to very distinct topologies. In Fig. 3a more detailed analysis and comparison to optimized and random networks is presented. All the evolved networks show degree distributions clearly distinct from random and optimized configurations, cf. Fig. 3a. The degree distributions of non-synchronized networks are skewed and while some nodes tend to become disconnected there is a clear tendency for hub formation. In contrast, for the synchrony enhanced networks the support of the distributions is narrow, where a clear peak for nodes of degree three stands out. These results agree with previous findings for synchrony enhancement for identical^33,34^ and non-identical^19,20^ oscillators. Also correlations between native frequencies and in-degree show clear patterns, see Fig. 3b. For the synchronized and optimized networks a positive correlation is observed such that oscillators with native frequencies far off the mean frequency have large in-degrees – as is required to draw them into the collective rhythm. However, note a small difference between the optimized and evolved networks for small native frequencies when the relationship reaches a plateau towards *ω* = 0 in one case and exhibits a sharp minimum in the other. The opposite correlation pattern holds true for non-synchronized networks, for which links are concentrated around oscillators with native frequencies close to the center. In spite of a small tendency towards disassortative degree mixing measured by *a* = −0.16 ± 0.04^32^ one concludes that the evolved non-synchronized networks have a core-periphery architecture (see also Supplementary Figs S6 and S7 for more detail). Further analysis of synchronized clusters shows that synchronization prevails within the core (see Supplementary Fig. S3), but the majority of the off-centre native frequency oscillators have too little input from the core and remain un-synchronized. Synchronized systems typically exhibit strong dis-assortative mixing by degree, which can be concluded from Fig. 3f in which we plot the dependence of average neighbor degrees on native frequencies combined with information in panel (b). Disassortative mixing by degree has been observed to be a hallmark of highly synchronizable networks before^35,36^, supporting our claim that competitive influence maximization tends to enhance synchronization. We note that mixing patterns are particularly strong in the competitively evolved networks (*a* = −0.73 ± 0.06 vs. *a* = −0.19 ± 0.05 for the optimized networks), hinting at further correlation patterns.

Indeed, non-trivial patterns are also found in correlations of native frequencies of linked oscillators. We illustrate them in panels (c) and (d) as average and variance of the distribution of native frequencies of nodes with given native frequencies. A richer picture is obtained by visualizations of the *ω* − *ω* correlation matrices in which is recorded the probability that a node of native frequency *ω*_1_ is linked in the evolved network to a node of native frequency *ω*_2_, c.f. Fig. 3g–i. Here the *x*-axis denotes the ‘control’ node label for the frequency, as it seeks to drag the collective frequency to it, whereas the *y* – axis denotes the ‘receive’ node label. As observed before^20,22,24^, arrangements are generally anti-correlated for synchrony-enhanced networks, see panels (g) and (i) in which most probability mass is concentrated in the 2nd and 4th quadrant, but have failed to evolve towards a clear pattern in the un-synchronized networks, c.f. panel (h). Correlation patterns are further distinct for competitively evolved and optimized networks. In the former case, nodes with native frequency *ω* almost exclusively receive input from other nodes with native frequency in a narrow band of frequencies around −sgn(*ω*)(1 − |*ω*|); within this, nodes with *ω* ≈ 0 in the center are controlled and controlling nodes with *ω* = ±1 at the boundary of the native frequency distribution (hence also the strong peak in variance in panel (d) and the plateau in the dependence of 〈*k*_in_〉 on *ω* in panel (b)). This connection pattern, which is also related to enhanced frequencies of reciprocated links (see Fig. 3e), arises from the particular way in which clusters of synchronized nodes can grow under the competitive rewiring dynamics. Such clusters initially mostly consist of oscillators in the center of the native frequency distribution, and then broaden out to include oscillators further and further away from the mean (see Supplementary Fig. S3 for more details).

This leads to the following picture of influence maximization. Oscillators generally tend to enhance their influence by linking to nodes with a large negative frequency distance. Further, nodes with many incoming links are more difficult to influence than nodes with lower in-degree. Thus, a node’s attractiveness to acquire new connections is a trade-off between its native frequency gap to the target and its in-degree. Consequently, starting from a synchronized seed cluster of nodes with close to zero native frequency, nodes within the cluster initially tend to link to nodes at the extreme end of the frequency distribution. Nodes not yet part of the synchronized cluster can enhance their influence in two ways. The first is by establishing connections to other nodes with maximum native frequency gap, even if these might not be strongly enough connected to the synchronized cluster to influence it. Alternatively, their influence can be enhanced by linking to nodes within the synchronized cluster, thus being able to change the average rhythm of this cluster. What emerges is a trade-off between frequency gap maximization and linking to large synchronized clusters, in that links to nodes in the synchronized cluster with maximized frequency gap are reciprocated. When in-degrees of the farthest off-center nodes have saturated, the next generation of close-to-center native frequency nodes recruited into the synchronized cluster will establish connections to nodes slightly off the extremes of the native frequency distribution, and so on, explaining the correlations shown in Fig. 3f.

### Regimes for different coupling strengths

**Figure 4:**
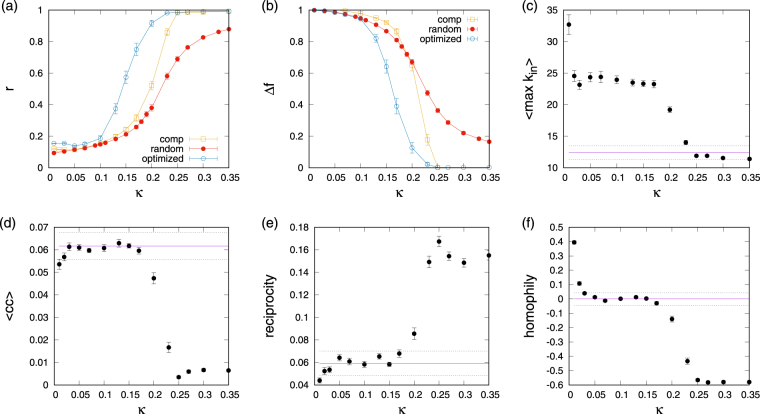
Properties of networks depending on the coupling strength *κ* for which they were evolved: (**a**) phase synchronization order parameter *r*, (**b**) frequency synchronization order parameter Δ*f*, (**c**) maximum in-degree, (**d**) clustering coefficient, (**e**) reciprocity, and (**f**) native frequency homophily. Points represent averages over at least 20 networks of size *N* = 100 with out-degree *k*_out_ = 6. The solid line in panels (c–f) gives expectations for an out-degree regular graph, dashed lines give an interval of one standard deviation around it.

Next we proceed with a more systematic exploration of the dependence of the evolved networks on the coupling strengths. A summary of results is reported in Fig. 4 which also includes comparisons to random networks in the absence of optimization. Panels (a) and (b) give results of the phase and frequency synchronization order parameters on the coupling strength; the remaining panels illustrate the change of various network properties with coupling. Noticing the two plateaus and the distinct very low coupling phase in panels (c–f) of 4 it becomes clear that three regimes of coupling strengths exist: (i) a regime of very low coupling *κ* ≤ 0.02 in which networks with very strong positive assortment by native frequency are formed, (ii) a regime of low to intermediate coupling 0.03 ≤ *κ* ≤ 0.17 in which core-periphery type networks as described above emerge, and (iii) a regime of fairly high coupling *κ* ≥ 0.2 in which competitive influence maximization enhances synchronization. In regime (i) due to large maximum in-degrees (cf. 4c) networks become very heterogeneous, short loops and triangles are avoided (cf. 4d,e), and the strongest hallmark of network organization becomes a preponderant tendency of oscillators of like native frequency to link to each other (cf. 4f). Networks in this regime have no clearly defined unique core. Network structures pertaining to regime (ii) and (iii) have been discussed above for the specific case of synchronizing and non-synchronizing networks evolved for *κ* = 0.23, but in Fig. 4 we notice that their properties are indeed typical for the corresponding regimes. Hence, in regime (ii) networks are distinguished by cores comprised of close-to-the-center native frequency oscillators, whereas the large peripheral parts of the network retain their close-to random organization. In regime (iii) hallmarks of network organization known to be associated with enhanced synchronization emerge. This rough subdivision of phases and the tendency for enhancement of synchronization with influence maximization in phase (iii) are not unique to uniform distributions of native frequencies. To ensure the robustness of our result, we also investigated Gaussian and bimodal distributions of native frequencies and find similar behaviour in these cases (see Supplementary Fig. S2 for details).

### Partial rewiring

**Figure 5:**
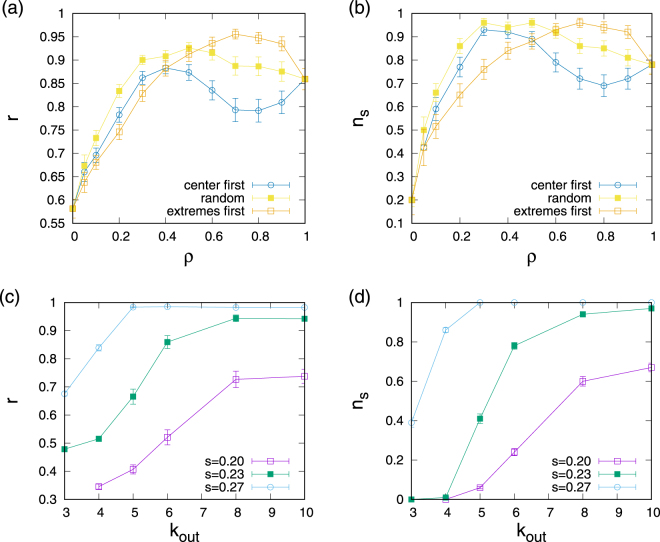
Dependence of synchronization properties of evolved networks on the number of influence maximizing oscillators and dependence on connectivity: (**a**) and (**b**) phase synchronization order parameter *r* and fraction of synchronized configurations *n*_*s*_ when only a fraction *ρ* of the oscillators takes part in the competitive influence maximization; (**c**) and (**d**) phase synchronization order parameter and fraction of synchronized oscillators for networks evolved for different connectivities *k*_out_. For (**a**) and (**b**) *N* = 100, *κ* = 0.2, *κ* = 0.23, *κ* = 0.27 and for (**c**) and (**d**) the coupling strengths were adjusted for each connectivity to ensure *r* ≈ 0.39 (for *κ* = 0.2), *r* ≈ 0.58 (for *κ* = 0.23), and *r* ≈ 0.77 (for *κ* = 0.27) without rewiring. Points represent averages over 100 independent runs, error bars give standard deviations.

It is also of interest to investigate situations in which not all Kuramoto agents participate in the rewiring process. Accordingly, we designed experiments in which only a fraction *ρ* of all oscillators is selected for active rewiring, i.e. only selected oscillators competitively rewire their out-connections whereas out-connections of all other oscillators remain fixed during the simulation. We also explore scenarios with a bias for oscillator selection: either by only selecting a fraction *ρ* of oscillators with native frequencies close to the center of the distribution or by selecting oscillators starting at the extremes of the distribution. Technically, this selection is achieved by ordering nodes by ascending absolute value of their native frequencies ${\{|{\omega }_{i}|\}}_{i=0}^{N-1}$ such that |*ω*_*i*_| ≥ |*ω*_*j*_| all *i* > *j*, and either setting the first *i* = 0, ..., *ρN* oscillators (center first) or the last oscillators *i* = (1 − *ρ*)*N*, ..., *N* (extremes first) active for rewiring. For maximum sensitivity to *ρ* experiments were carried out in the transition region with *κ* = 0.23. Results are illustrated in Fig. 5a in which the dependence of the phase synchronization order parameter *r* on the density of active oscillators *ρ* is shown. Since the distribution of outcomes of the competitive influence maximization is bimodal (see Fig. 2f), we also define a fraction of synchronizing configurations by counting the fraction *n*_*s*_ of all runs in which networks have achieved *r* > 0.7 (note that any threshold in between the high and low synchrony peaks can be chosen here) and plot *n*_*s*_ vs *ρ* in Fig. 5b. Inspecting Fig. 5a,b several observations stand out. First, substantial enhancement of synchrony can already be achieved when only a small fraction of oscillators participates in the rewiring. Second, the relationship between *ρ* and measures of synchronization is generally not monotonic: a certain share of oscillators participating is required to achieve high synchronization, but effects of competition when too many oscillators participate has a slightly detrimental effect on the level of synchronization achieved. Third, we notice that biased selection of rewiring oscillators influences outcomes. If oscillators with native frequencies close to the center are preferentially chosen, high levels of synchrony can be achieved with fewer actively participating oscillators, but the level of achieved phase synchronization is lower, since some high native frequency oscillators could not be recruited into the synchronized cluster. In contrast, biasing towards off the center oscillators for rewiring can boost synchronization beyond random selection, but a larger fraction of oscillators (compared to centre-first bias) needs to be selected to achieve this.

### Dependence on connectivity

As a last point we address the dependence of results on the connectivity of networks, where up till now *k*_out_ = 6 has been used. For this purpose, experiments are repeated for out-degrees ranging between *k*_out_ = 3 and *k*_out_ = 10 where saturation is observed. To enable sensible comparisons between different connectivities, coupling strengths for each connectivity were tuned to give the same degree of phase synchronization for out-degree regular random networks as for the reference case of *k*_out_ = 6. In this way, for each connectivity experiments started with the same initial degree of phase synchronization. Then, competitive influence maximizing rewiring was carried out and the stationary values of *r* and the fraction of synchronizing configurations *n*_*s*_ were recorded. Results are displayed in Fig. 5c,d, where we notice that influence maximization increasingly leads to concurrent enhancement of synchronization the larger the connectivity of the network. In fact, we find transition-like behaviour: for low connectivities no enhancement of synchronization through influence maximization can be achieved, whereas for larger connectivities and large enough coupling strengths almost all configurations allowed for influence maximization to substantially boost synchronization.

## Discussion

In this paper we have considered a process of network formation between non-identical Kuramoto oscillators based on individual oscillators competitively rewiring their out-connections with an aim to maximize their influence on the collective. For this we identified oscillators’ native frequencies with their goals and subsequently considered a scenario in which each oscillator tries to arrange its connections with the aim to make the system synchronize to its native frequency. We have shown that outcomes of such competitive influence-maximizing rewiring depend on the coupling strength and connectivity. Regimes for low and intermediate coupling exist, in which networks are rewired towards core-periphery architectures in which at least part of the close-to center oscillators are recruited to a frequency locked cluster. More interestingly, however, for all the scenarios we investigated, a regime exists in which competitive influence between agents of very widely divergent goals drives the system toward a synchrony-enhanced state. We find that typical network configurations in this state bear many of the hallmarks of network organization previously associated with synchrony-enhancement^20,22,24,29^. Additionally, however, networks also carry a signature resulting from the growth process of the largest synchronized cluster, namely particular correlations of native frequencies of adjacent oscillators.

Traditionally, as pioneered by^37^ influence maximization has been typically considered in the very different context of *one* agent attempting to convert opinions of sets of agents with binary opinions in probabilistic settings. In this paper we are the first to consider the other extreme of the spectrum: a set of *many* agents with widely divergent goals competitively attempting to maximize their individual impact on the system. As we show for the particular case of non-linearly diffusively coupled Kuramoto agents, such a process can actually enhance consensus formation. Interestingly, this is already the case if only a small fraction of agents take part in the rewiring and can be optimized if only a certain fraction of agents is active.

We believe our results are of importance in at least two respects. First, to our knowledge, synchrony-enhancement has mostly been considered in a context where a central planner has the power to arrange system structure^18,20,23^. Here we show how enhanced synchrony might arise as a by-product of influence maximization which might be a more realistic pathway for systems comprised of many self-interested heterogeneous agents. Second, results of this work would be of interest in the wider context of consensus formation in social dynamics^38^. One wonders if our particular results in the context of the Kuramoto model can be generalized to typical models of consensus formation, which we believe is an interesting direction for future work.

## Electronic supplementary material

Supplementary information
