# Supplementary material for: Competitive influence maximization and enhancement of synchronization in populations of non-identical Kuramoto oscillators
Source: Sci Rep. 2018 Jan 15;8:702. doi: 10.1038/s41598-017-18961-z (PMC5768883; doi:10.1038/s41598-017-18961-z)
Supplement: Supplementary file 1 — Supplementary information [file 41598_2017_18961_MOESM1_ESM.pdf]

Supplement to the main paper: Competitive influence maximization and enhancement of synchronization in populations of non-identical Kuramoto oscillators

Markus Brede[1,*], Massimo Stella[1,2], and Alexander C. Kalloniatis[3]

[1]University of Southampton, Electronics and Computer Science, Southampton, SO171BJ, UK
[2]Fondazione Bruno Kessler, Italy
[3]Joint and Operations Analysis Division, Defence Science and Technology Group, Canberra, 2600, Australia
[*]brede.markus@gmail.com

ABSTRACT

In this supplement to the main paper "Competitive influence maximization and enhancement of synchronization in populations of non-identical Kuramoto oscillators" additional results to support the conclusions of the main paper are presented.

Additional results

This supplement presents some additional results in support of the conclusions of the main paper.

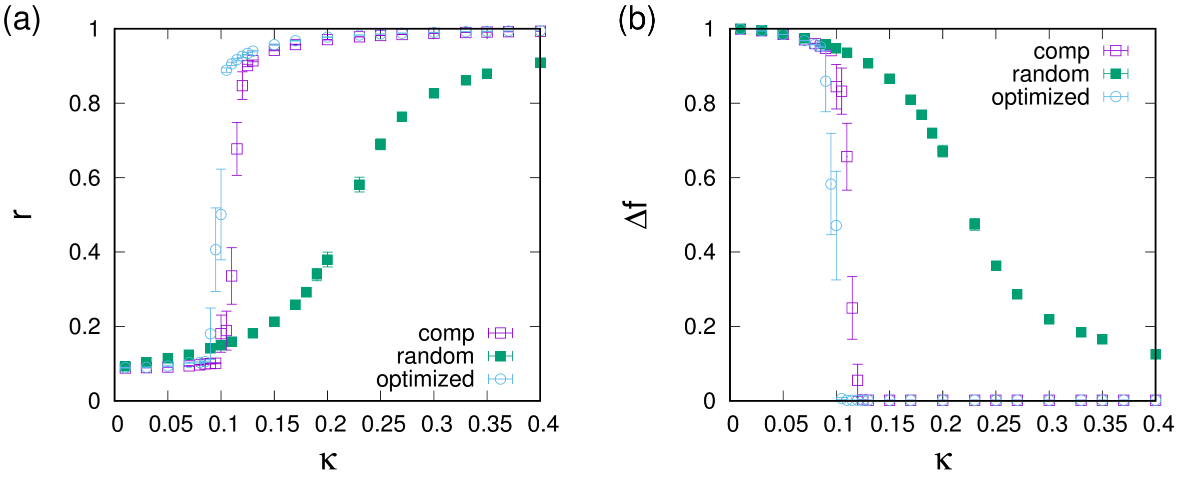

Figure 1. Dependence of the order parameters r and Δf on the coupling strength for networks of size $N = 100$ with out-degree $k_{\text{out}} = 6$ evolved for $\kappa = 0.27$: competitively evolved networks, random networks, and optimized networks (see legends). Data points represent averages over 100 independent runs and error bars give the estimated standard errors.

Full dependence of synchronization on coupling strengths. Supplementary Figure S1 gives results to demonstrate that configurations that have either been competitively evolved or optimized for specific coupling strengths are indeed synchrony-optimal over a wider range of coupling strengths. For the experiments presented in Supplementary Fig. S1 networks were evolved for $\kappa = 0.27$ and we then explored the synchronization behavior of these networks over a range of coupling strengths. Even though the networks have been evolved for $\kappa = 0.27$, enhanced synchronization is observed over a large interval of coupling strengths and the transition towards synchronization on the evolved networks occurs markedly earlier than on random graphs. This manifests itself in larger degrees of phase synchronization (panel (a)) and better frequency synchronization (panel (b)) than random networks for $\kappa \geq 0.1$. In contrast, slightly worse synchronization behavior is observed for evolved networks for $\kappa < 0.1$. Note also that the competitively evolved networks show only slightly worse synchronization behavior than the

optimized networks.

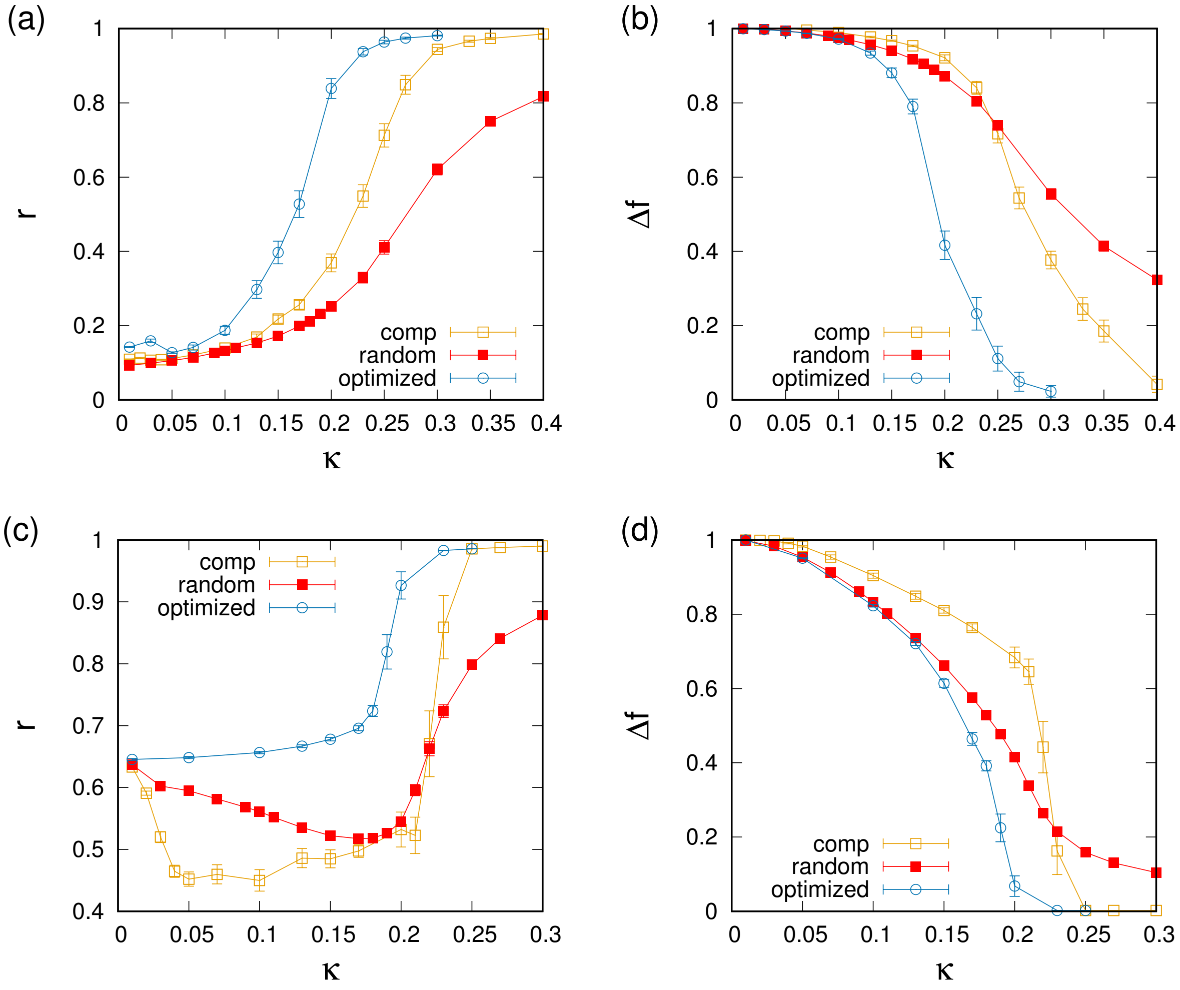

Figure 2. Comparison of dependence of phase and frequency synchronization order parameters r and Δf on coupling strength κ for competitively evolved, out-degree regular random and optimized networks for Gaussian native frequency distributions with mean zero and standard deviation $\sqrt{2/3}$ of native frequencies (top row), and for a bimodal distribution of native frequencies with equal peaks at $\omega = -1$ and $\omega = 1$. The networks are of size $N = 100$, $k_{\mathrm{out}} = 6$, and data points represent averages over at least 20 independent runs. When not visible, error bars are less than the size of the symbols.

In Supplementary Fig. S2 we generalize the above procedure for $\kappa \neq 0.27$: networks were evolved for *varying* coupling strengths κ and then measures of phase and frequency synchronization computed with the evolved configurations are presented as a function of that coupling strength. The analysis in the main paper is focused on uniform distributions of native frequencies drawn from $[-1, 1]$; here we show that qualitatively similar behavior holds also for other distributions of native frequencies. The cases analyzed in Supplementary Fig. S2 give data for Gaussian distributions (panels (a) and (b)) with standard deviation equal to that of the uniform distribution between -1 and 1, and for the extreme case of a bimodal distribution of native frequencies $P(\omega) = 0.5\delta(\omega - 1) + 0.5\delta(\omega + 1)$ with peaks at $\omega = -1$ and $\omega = 1$. We note that in both situations a regime exists in which the coupling is large enough such that synchronization is enhanced by competitive influence maximization compared to random graphs; this is the case for $\kappa \geq 0.2$ for the Gaussian case and for $\kappa \geq 0.23$ for the bimodal case.

Alternative frequency distributions. To complement the discussion of the dependence of network properties on the coupling strength used for evolution, we also present the analysis for Gaussian and bimodal frequency distributions which corresponds to the analysis for uniform frequency distribution in Fig. 4 of the main paper. The results are given in Supplementary Fig. S3. Qualitatively similar results are evident for Gaussian (top row of panels) and bimodal (bottom row of panels) native frequency distributions; only small numerical differences occur in, for example the sharpness of the transitions between regimes. With the exception of panels (b) and (f), three regimes are evident in all cases.

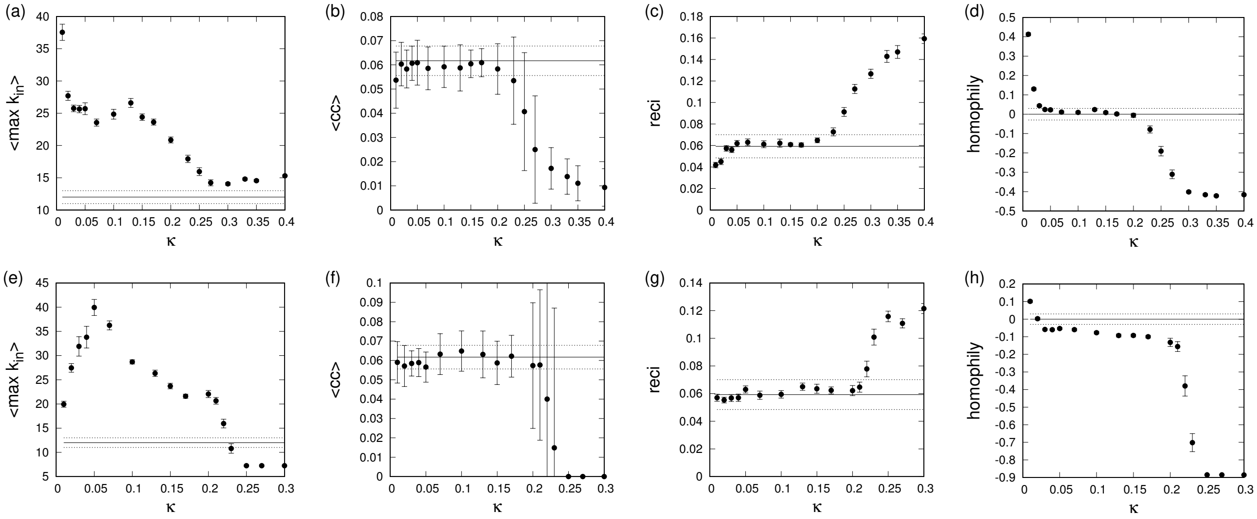

Figure 3. Network statistics as a function of the coupling strength κ for which the networks were evolved with competitive influence maximization: (a),(e) maximum in-degree, (b),(f) clustering coefficient, (c),(g) reciprocity, (d),(h) native frequency homophily. The top row represents networks evolved with a Gaussian native frequency distribution and the bottom row gives networks evolved with a bimodal native frequency distribution (see Supplementary Fig. S2 for details of the parameter settings). Data points represent averages over at least 20 independent runs.

Calculating synchronized clusters. In Supplementary Fig. S4 analysis of the evolution of synchronized clusters during the process of competitive influence maximization is presented. For this purpose we define an undirected graph G made up of all nodes of the evolved directed network Γ and containing only links (i,j) of Γ if $\left|f_i - f_j\right| < \delta$ where the $f_i = \langle \dot{\phi}_i \rangle_{[T/2,T]}$ are the time averaged instantaneous frequencies of the oscillators and $\delta = 0.002$ represents a threshold determined by the maximum frequency difference that can be resolved with the numerical integration scheme. Synchronized clusters are then defined as connected components of G and we measure the average size of the connected component N_s to which a node belongs. To track then the evolution of synchronized clusters, networks are evolved until a certain degree of phase synchronization r is achieved and configurations, as just defined, are recorded and analyzed. Larger values of r correspond to later stages of the evolution. Thus, by comparing synchronized clusters for increasing values of r the growth of synchronized clusters can be understood.

In Supplementary Fig. S4a we see a clear dependence of N_s on ω: nodes close to the center of the native frequency distribution tend to be in larger synchronized clusters than nodes at the boundary of the native frequency distribution. In that panel we also see that, under competitive influence maximization, synchronization starts with a small cluster of synchronized oscillators with close-to-center native frequencies, which then expands by more and more off-center oscillators being recruited. Oscillators at the boundaries of the native frequency distribution are recruited last.

Panel (b) of 4 shows the dependence of N_s on ω for the non-synchronizing networks evolved for $\kappa = 0.23$. The result complements discussion in the main text and points out that also in the non-synchronized cases small synchronized clusters exist but only include nodes with close to the mean native frequency.

Dependence on number of rewiring oscillators. Complementing results shown for $\kappa = 0.23$ in Fig. 5 of the main paper, Supplementary Fig. S5 here gives additional data for $\kappa = 0.20$ (panel (a) and (b)) and $\kappa = 0.27$ (panels (c) and (d)). In the figures we analyze experiments in which only a fraction ρ of all oscillators has taken part in the influence maximization. These oscillators have either been selected at random, or biased, by either only choosing oscillators from the center or from the boundaries of the native frequency distribution (see main paper for details). Here we note that in all cases a non-linear dependence of the achieved degree of phase synchronization r and of the number of synchronizing configurations n_s on ρ is found. For relatively low coupling ($\kappa = 0.2$) the observed patterns are qualitatively the same as described in the text pertaining to Fig. 5 in the main paper. For large coupling ($\kappa = 0.27$) only a very small fraction of oscillators is needed to achieve close to perfect synchronization, and for $\rho > 0.05$ all configurations can achieve full synchronization. In this case the dependence of synchronization on ρ is monotonic.

In principle, considering the average degree of phase synchronization r as shown in Supplementary Fig. S5 (a) and (c) high synchronization can be achieved in two ways: Either (i) by individual synchronizing configurations each achieving a higher degree of synchronization or (ii) by more configurations being able to synchronize, but without the synchronizing configurations reaching a higher degree of synchronization than in (i). In Supplementary Fig. S5 (c) and (d) a scenario is investigated where all

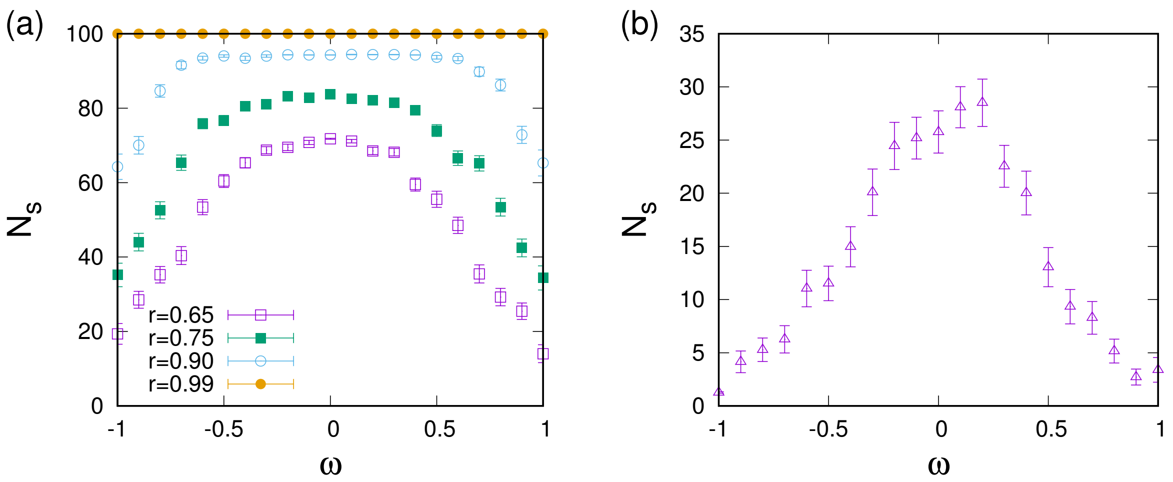

Figure 4. Dependence of the average size of a synchronized cluster N_s to which a node belongs on its native frequency. In (a) snapshots were recorded during the competitive evolution for $\kappa = 0.27$ when $r = 0.65$, $r = 0.75$, $r = 0.90$ and $r = 0.99$ were reached. The figure illustrates that synchronized clusters start to grow with nodes of native frequencies close to the mean and then gradually spread out to include nodes with native frequencies farther off the mean, and eventually all nodes are included. In (b) dependence of N_s on ω for the un-synchronized networks (as evolved for $\kappa = 0.23$) is visualized. Systems are of size $N = 100$ with $k_{\text{out}} = 6$.

configurations can reach synchronization and we see that the effect of (i) is rather small. This suggests that the main influence of different oscillator choices for active rewiring is effect (ii). In other words: for low coupling, partial (and biased) selection of oscillators that can carry out rewiring can substantially improve chances that a system can evolve towards full synchronization.

We also note that for high coupling, even though there are no large differences between random and biased selection of active oscillators, preferential inclusion of oscillators with native frequencies close to the boundary of the distribution can achieve best synchronization.

Core-periphery structure. As reported in the main text, in non-synchronized networks links are concentrated around oscillators with native frequencies close to the center, thus hinting at a core-periphery network structure. In order to quantitatively test the presence of network cores, we adopted the methodology of Ma and Mondragón[1], which is similar to detecting a rich-club effect in the allocation of edges in a network but: (i) It does not display large fluctuations for small networks and (ii) It can also identify which nodes belong to the core. Notice that compared to other core-periphery measures such as Holme's[2], the rich-core measurement does not rely on strict requirements such as identifying cores with k-cores, which would be problematic for networks as small as the ones analyzed in the main text.

Ma and Mondragón's methodology for directed networks ranks nodes in descending order of their in-degree. For every node i of rank r_i, the number k_i^+ of its links to nodes of higher rank $r_j < r_i$ is counted. Plotting k_i^+ as a function of the ranking provides a profile of the way nodes allocate their links. Increasing (decreasing) trends of k^+ indicate a concentration of edges among higher (lower) rank nodes, so that a rich core is present when k^+ displays a well defined maximum[1] K^+ compared to networks generated from a suitable null model.

Results of the rich core analysis for non-synchronized networks are reported in Supplementary Fig. S6 (a) and contrasted against a reference null model (out-degree regular random graphs) in Supplementary Fig. S6 (b). Non-synchronized networks display a well defined maximum in the number of links directed to higher rank (e.g. higher in-degree) nodes: The 43 nodes with the highest in-degree tend to share links among themselves rather than with lower in-degree nodes, suggesting the presence of a network core. Notice also that the remaining nodes tend to either allocate edges to the core or among themselves, thus leading to decreases in k^+ and indicating the presence of a network periphery. This core-periphery structure is not present in out-degree regular random graphs, considered as reference null models and obtained by random rewiring of out-links. As evident from Supplementary Fig. S6 (b), the reference model does not display a well defined maximum K^+ and a decreasing trend in k^+, thus it lacks a core-periphery structure.

A comparison of native frequencies for nodes within and outside of the identified rich cores is reported in Supplementary Fig. S7. While both "core" and "peripheral" nodes have median native frequencies close to 0, interquartile ranges, accounting for 50% of the distribution of frequencies, are considerably narrower for core nodes. This further confirms the finding discussed in the main text that core nodes have native frequencies closer to the center.

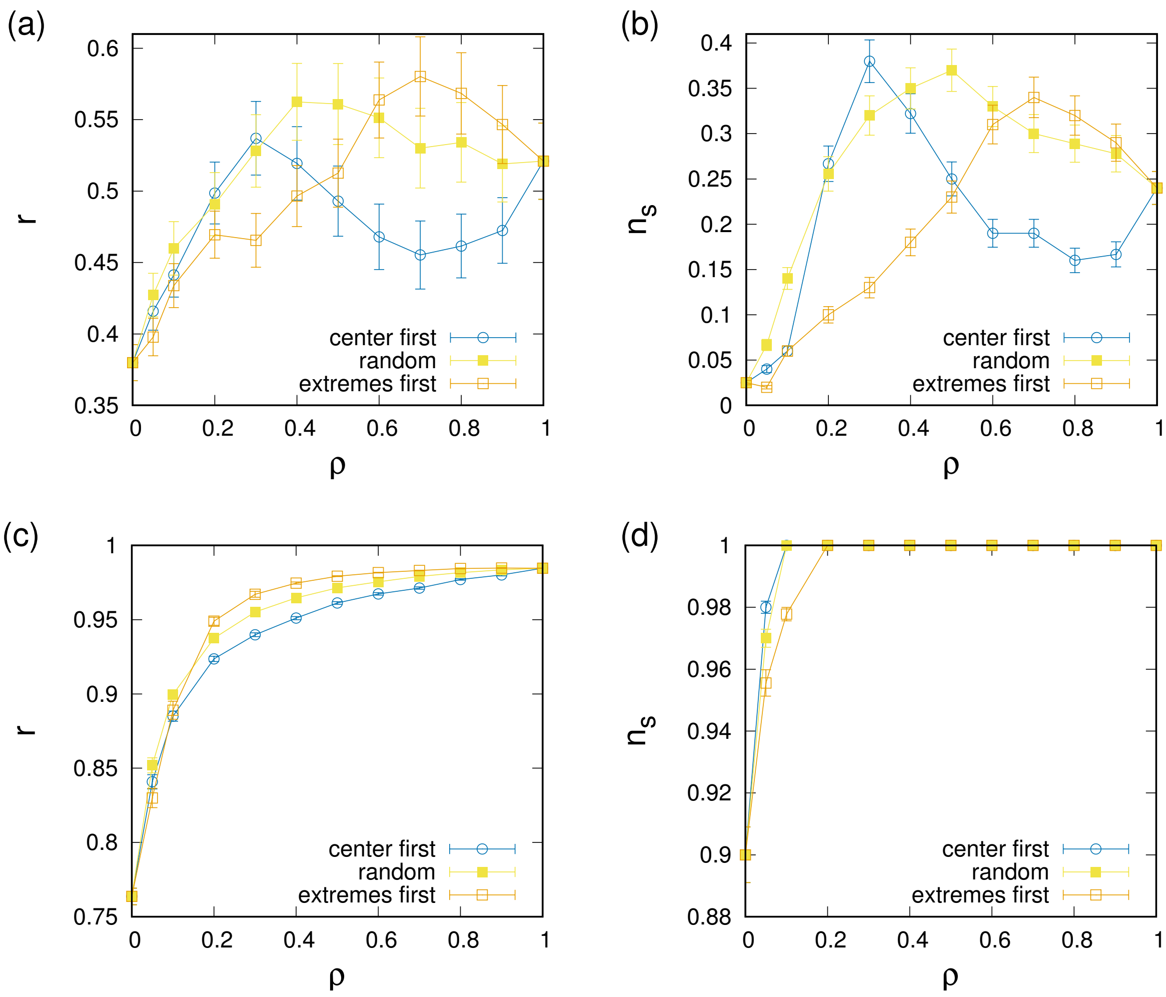

Figure 5. Dependence of synchronization properties of evolved networks on the number of influence maximizing oscillators when only a fraction ρ of the oscillators takes part in the competitive influence maximization. (a) and (c): phase synchronization order parameter r and (b) and (d): fraction of synchronized configurations n_s. (a) and (b) give data for networks evolved at $\kappa = 0.20$ and (c) and (d) for networks evolved for $\kappa = 0.27$. All data points represent averages over 100 independent runs. The data in this figure complement results presented in Fig. 5 in the main text and demonstrate that the findings discussed in the main text are robust.

The above findings provide quantitative confirmation of the claims of core-periphery structure in non-synchronized networks reported and discussed in the main text.

References

1. Ma, A. & Mondragón, R. J. Rich-cores in networks. *PloS one* **10**, e0119678 (2015).

2. Holme, P. Core-periphery organization of complex networks. *Phys. Rev. E* **72**, 046111 (2005).

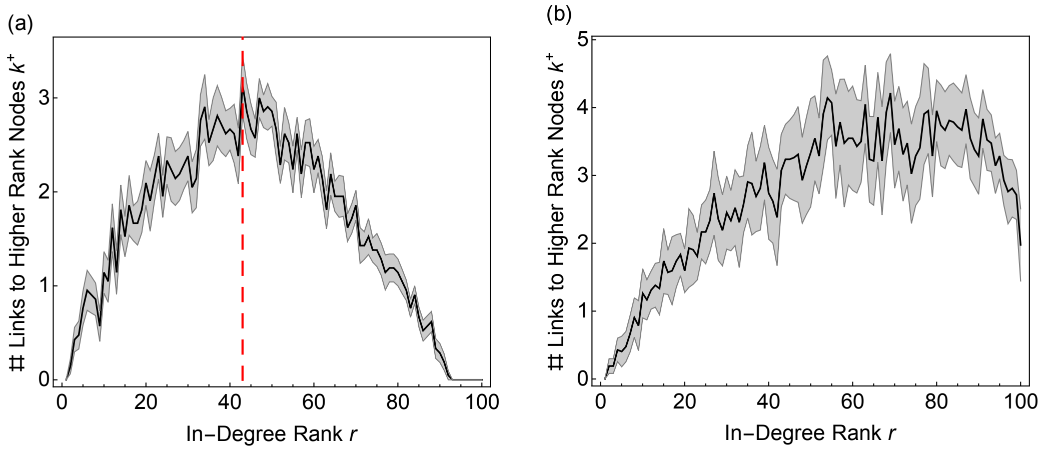

Figure 6. Rich core profiles for non-synchronized networks (a) and for randomized out-degree regular networks, used as reference null models. A profile ranks nodes according to their in-degree and it indicates the number of links to higher rank nodes. Results are averaged over 21 network configurations. The black line indicates the average while he gray overlay indicates one standard deviation from the average. A well-defined maximum (red line) is present in non-synchronized networks, indicating the presence of a rich core with roughly 43 nodes in it. The reference null model does not display any well defined maximum, suggesting a lack of core-periphery structure. The data in this figure complement results presented in Fig. 3 of the main text about the core-periphery structure of non-synchronized networks.

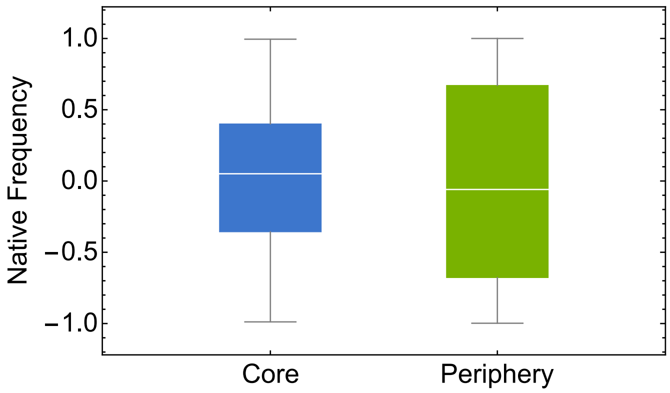

Figure 7. Box plot of the native frequencies for nodes identified within the rich cores (blue, left) and outside of them (green, right) averaged over 21 non-synchronized networks. A comparison of the interquartile ranges (i.e. boxes) confirms that nodes in the network cores are more concentrated around the center.
